# Circular RNA expression profiles reveal that hsa_circ_0018289 is up-regulated in cervical cancer and promotes the tumorigenesis

**DOI:** 10.18632/oncotarget.21257

**Published:** 2017-09-23

**Authors:** Ya-Li Gao, Ming-Yun Zhang, Bo Xu, Li-Jie Han, Shou-Feng Lan, Ju Chen, Yu-Jin Dong, Li-Li Cao

**Affiliations:** ^1^ Department of Radiotherapy, Cangzhou Central Hospital, Hebei 061001, China; ^2^ Department of Radiotherapy, Zibo Central Hospital, Shandong 255020, China

**Keywords:** cervical cancer, circular RNA, hsa_circ_0018289, miR-497, tumorigenesis

## Abstract

Circular RNAs (circRNAs) are a type of non-coding RNAs that have been identified as critical regulators in various diseases, especially in cancers. However, the expression profiles and functions of circRNAs in cervical cancer are still unclear. In present study, human circRNAs microarray were performed to screen the circRNAs expression in cervical cancer tissue. Microarray analysis revealed 45 significantly expressed circRNAs with 4 fold change. Among these up-regulated circRNAs, hsa_circ_0018289 was validated to be significantly up-regulated in 35 pairs of cervical cancer tissue compared with adjacent normal tissue and cell lines. Loss-of-function experiments revealed that, *in vitro* and *in vivo*, hsa_circ_0018289 knockdown inhibited the proliferation, migration and invasion of cervical cancer cells. Via bioinformatics prediction program and luciferase reporter assays, hsa_circ_0018289 was observed to directly bind to miR-497. Taken together, the results indicate that hsa_circ_0018289 plays important role in cervical cancer proliferation, migration and invasion, suggesting the miRNA ‘sponge’ of hsa_circ_0018289 and its oncogenic role on cervical cancer tumorigenesis.

## INTRODUCTION

Cervical cancer is one of the most common gynecologic tumors and accounts for large percentage of tumor associated death worldwide [1, 2]. Every year, there are hundreds or thousands of new cervical cancer patients reported in developing country or developed country [3]. Presently, although various methods have attempted for the treatment of cervical cancer, including surgery, radiotherapy and chemotherapy, the high lymphatic metastasis and distant metastases still induce the poor prognosis and low survival rate [4]. It must be admitted that multifarious molecular and pathways are involved in the carcinogenesis of cervical cancer, such as regulating proteins and non-coding RNAs (ncRNAs). In spite of this, the underlying functional molecular and regulation mechanisms in cervical cancer tumorigenesis are still unclear and need to be fully identified [5].

Circular RNAs (circRNAs) is an emerging type of ncRNAs without protein translation capacity [6–8]. CircRNAs is characterized by covalently closed loops without 3’- and 5’- end, which is absolutely different from linear RNA [9]. Compared to microRNAs (miRNAs) and long non-coding RNAs (lncRNAs), circRNAs have more stable construction and sequence conservation to resist the digestion of enzyme. Thus, circRNAs could much easier to be accumulated in cytoplasm or interstitial fluid, presenting higher concentration than corresponding linear RNAs. Increasing evidences have indicated that circRNAs take part in the regulation of tumorigenesis, including genesis, differentiation, migration and metastasis [10]. For instance, in hepatocellular carcinoma, ciRS-7 is significantly correlated with the clinicopathological characteristics of HCC patients and acts as an independent risk factor of hepatic microvascular invasion [11]. Besides, due to the stable expression and abundance, circRNAs could also act as valuable diagnostic marker for cancer detection. For instance, hsa_circ_0005075, a new identified circRNA, is involved in the progression of hepatocellular carcinoma and could act as a diagnostic marker [12].

MicroRNAs (miRNAs) are a kind of noncoding RNA with about 18-22 nucleotides. Usually, miRNAs participate in post-transcriptional regulation by targeting the 3’-UTR region of target genes. Plentiful researches have revealed the important role of miRNAs in various diseases. In cervical cancer, hundreds of miRNAs have been testified to regulate the proliferation, migration, invasion, and apoptosis. MiR-497 has been identified as tumor suppressor and inhibits the proliferation, migration and invasion of retinoblastoma, cervical cancer cells [13, 14].

In present study, our team screened the circRNAs expression profiles in cervical cancer tissue using human circRNA microarray assay, and ultimately identified a significantly overexpressed hsa_circ_0018289. Hsa_circ_0018289 is located at chr10:46968580-46969453 with 348 spliced length. Series of functional experiments revealed the important role of hsa_circ_0018289 on cervical cancer tumorigenesis. Besides, we also detected the interaction of hsa_circ_0018289 with miR-497. These finding provides valuable assistance for the prevention and treatment of cervical cancer.

## RESULTS

### CircRNA expression profile in cervical cancer tissue

**Figure 1:**
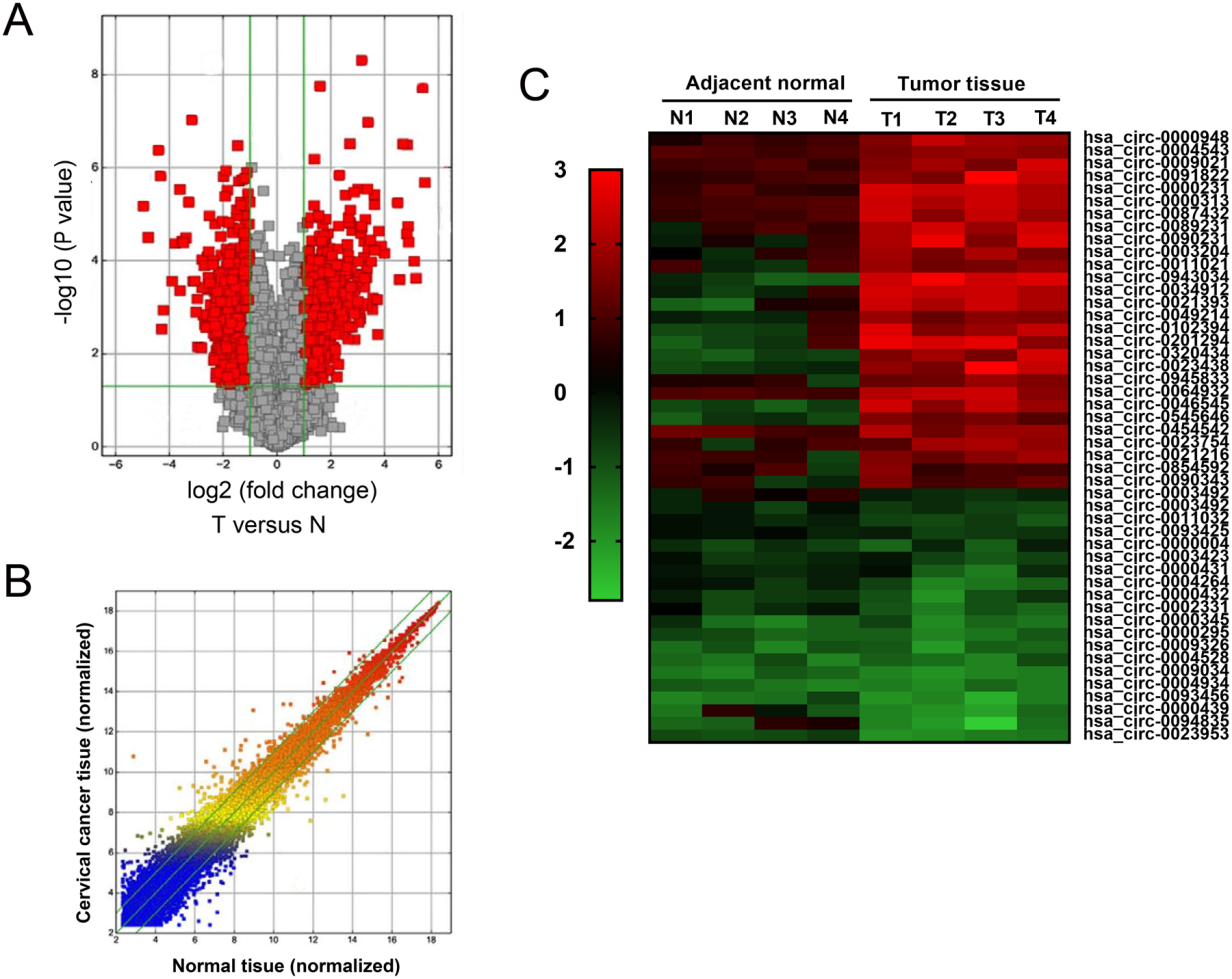
CircRNA expression profile in cervical cancer tissue **(A)** Volcano plot revealed the differently expressed circRNAs in cervical cancer tissue compared to adjacent normal tissue. **(B)** Scatter plot showed the differently expressed circRNAs. **(C)** Heat map showed the 45 significantly expressed circRNAs with 4 fold change (P<0.01) were presented, including 19 downregulated and 26 up-regulated circRNAs. Red indicated the upregulated expression with high fold-change and green indicated the downregulated expression with low fold-change.

In initial stage of experiments, 4 pairs of cervical cancer tissue and adjacent noncancerous tissue were performed for circRNA expression profile. Scatter plot and volcano plot revealed that total 393 dysregulated circRNAs with 2 fold change (P<0.01) were screened (Figure 1A). Heat map showed 45 significantly expressed circRNAs with 4 fold change (P<0.01) were presented, including 19 downregulated and 26 up-regulated (Figure 1B, 1C). This was the first time to reveal the circRNA expression profiles in cervical cancer tissue compared to normal tissue. These aberrantly expressed circRNAs might participate in cervical cancer tumorigenesis and play important role for tumor occurrence and development, providing plentiful potential functional circRNAs in cervical cancer carcinogenesis.

### Hsa_circ_0018289 was up-regulated in cervical cancer tissue and cells

**Figure 2:**
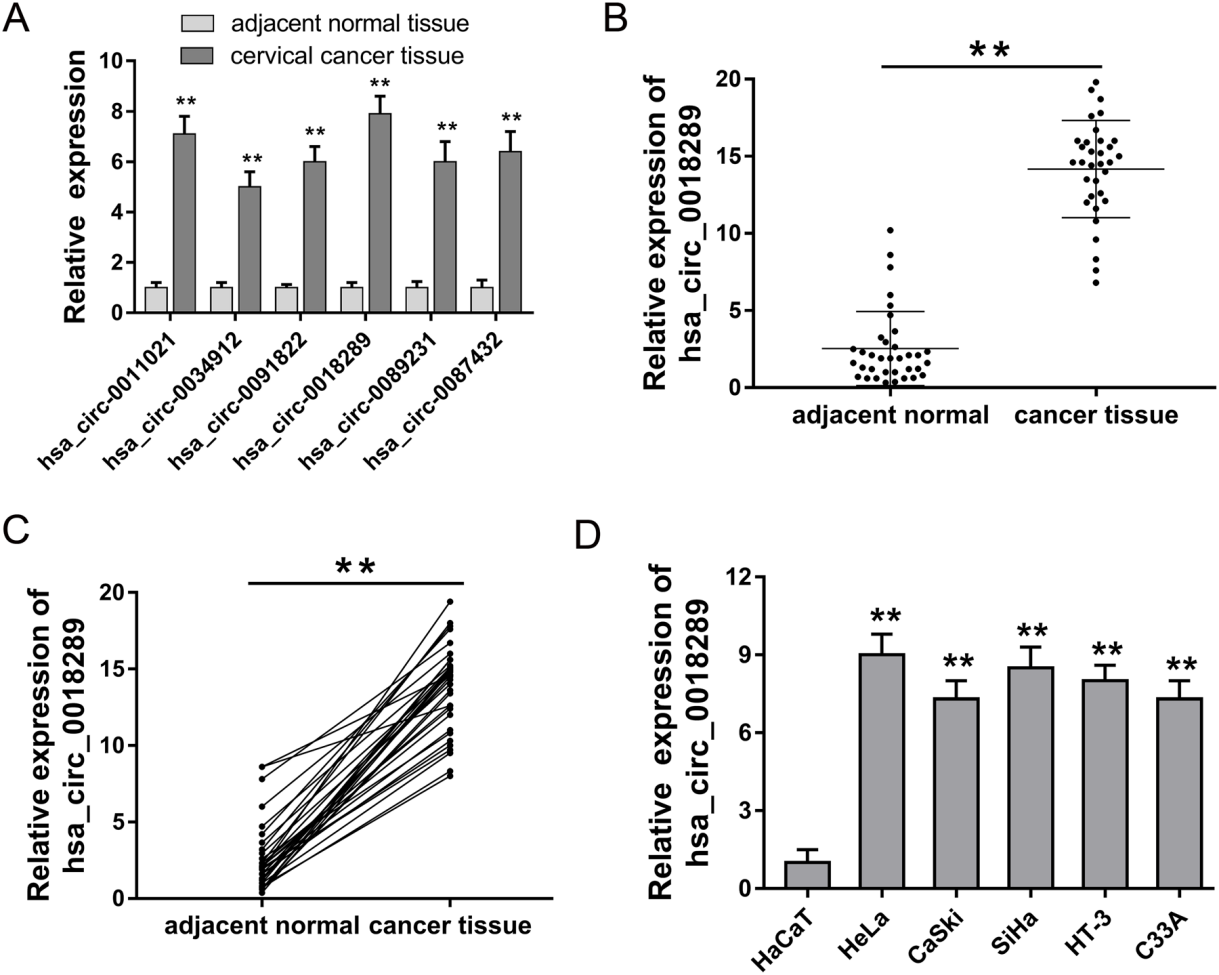
Hsa_circ_0018289 was up-regulated in cervical cancer tissue and cells **(A)** Six circRNAs were randomly selected to validate using RT-PCR, showing the significant overexpression of candidate circRNAs. **(B)** Hsa_circ_0018289 expression was validated in 35 pairs of cervical cancer tissue and adjacent normal tissue. **(C)** Hsa_circ_0018289 expression was upregulated in 94.3% (33/35) cervical cancer tissues. **(D)** Hsa_circ_0018289 expression was validated to be upregulated in cervical cancer cells compared to human epidermal cells. Data was represented as mean ± SEM. *P<0.05, **P<0.01 represents statistical differences calculated.

CircRNA microarray assay revealed the expression profiles of aberrantly expressed circRNAs in cervical cancer tissue compared to normal tissue. Among these up-regulated circRNAs, 6 circRNAs were randomly selected and validated using RT-PCR, showing the significant overexpression of candidate circRNAs (Figure 2A). Hsa_circ_0018289 was one of the up-regulated candidate circRNAs, and its expression was validated in 35 pairs of cervical cancer tissue and adjacent normal tissue (Figure 2B). Besides, hsa_circ_0018289 expression was upregulated in 94.3% (33/35) cervical cancer tissues compared with their adjacent non-tumor tissues (Figure 2C). In cervical cancer cells, hsa_circ_0018289 expression was also upregulated up compared to human epidermal cells (Figure 2D). Taken together, results confirmed the up-regulated expression of hsa_circ_0018289 in cervical cancer tissue and cells, suggesting a potential functional circRNAs in cervical cancer tumorigenesis.

### Hsa_circ_0018289 knockdown inhibited cervical cancer cells proliferation

**Figure 3:**
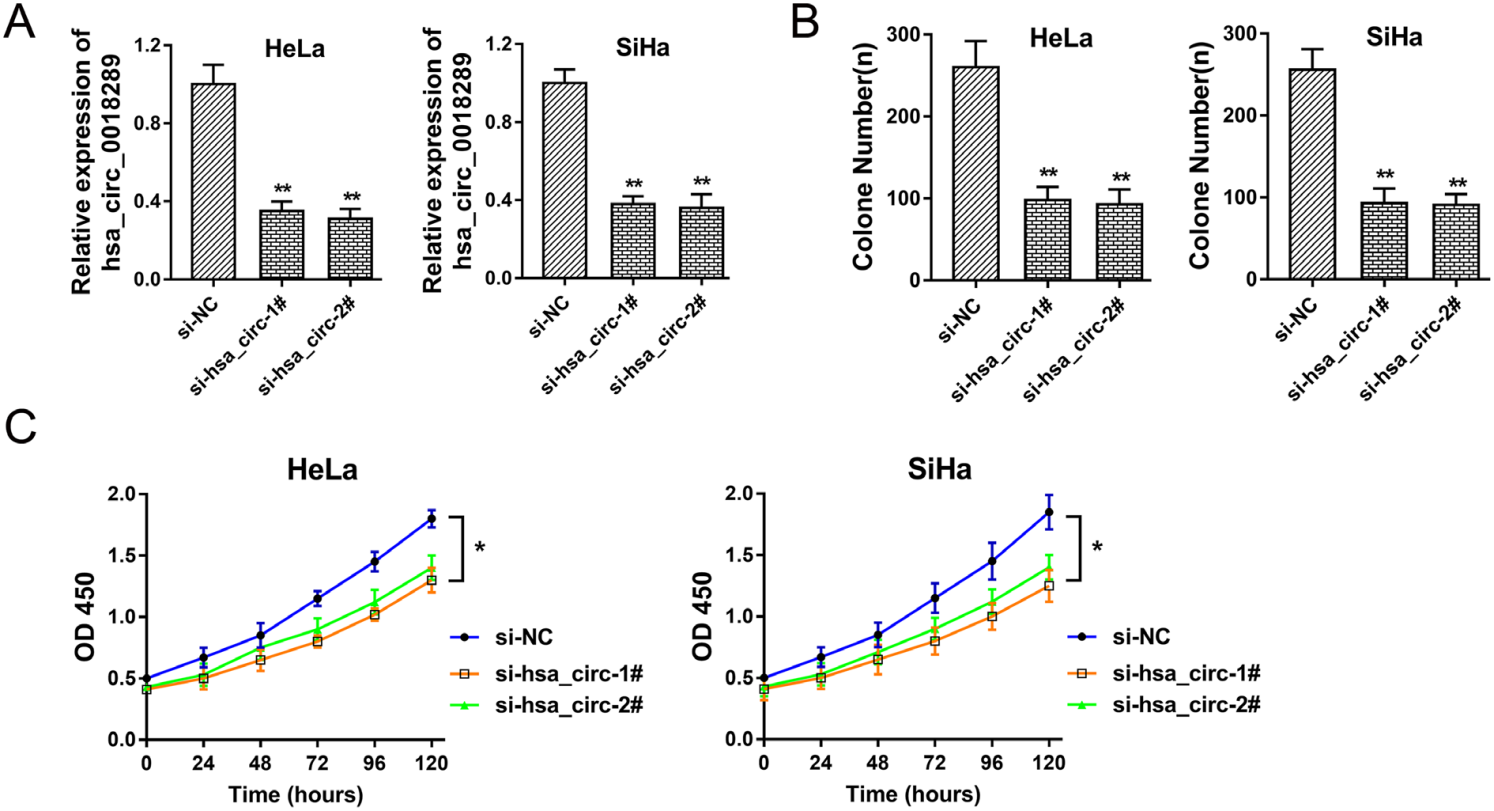
Hsa_circ_0018289 knockdown inhibited cervical cancer cells proliferation **(A)** Specially designed interfering oligonucleotides targeting hsa_circ_0018289 were synthesized to knock down hsa_circ_0018289 expression in HeLa and SiHa cells. **(B)** Colony formation assay showed the clone number in hsa_circ_0018289 knockdown and control group. **(C)** CCK-8 assay showed the proliferation ability in hsa_circ_0018289 knockdown and control group. Data was represented as mean ± SEM. *P<0.05, **P<0.01 represents statistical differences calculated.

Hsa_circ_0018289 was one of the significant up-regulated circRNAs in cervical cancer tissue and cells. To investigate the role of hsa_circ_0018289 in cervical cancer carcinogenesis, we performed loss-of-function experiments for hsa_circ_0018289 *in vitro*. Specially designed interfering oligonucleotides targeting hsa_circ_0018289 were synthesized to knock down hsa_circ_0018289 expression in HeLa and SiHa cells (Figure 3A). Colony formation assay showed that hsa_circ_0018289 knockdown could decrease clone number compared to control group (Figure 3B). Moreover, CCK-8 assay showed that hsa_circ_0018289 knockdown significantly suppressed the proliferation ability compared to control group (Figure 3C). In summary, results showed that hsa_circ_0018289 knockdown could inhibit the proliferation of cervical cancer cells *in vitro*.

### Hsa_circ_0018289 knockdown inhibited cervical cancer cells migration and invasion

**Figure 4:**
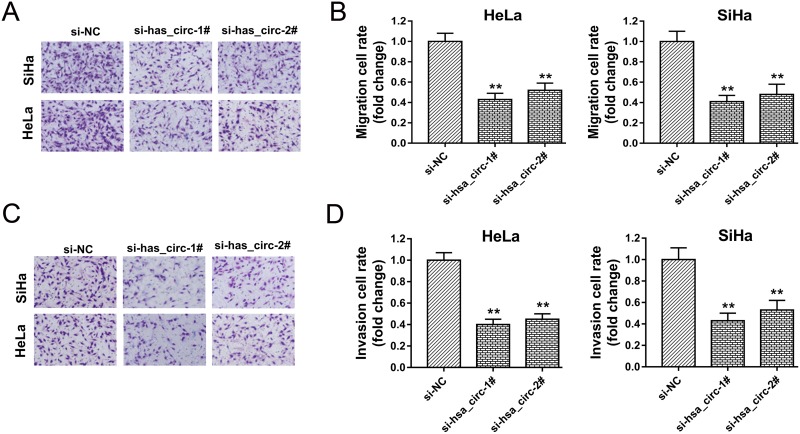
Hsa_circ_0018289 knockdown inhibited cervical cancer cells migration and invasion **(A)** Representative migrative images of HeLa and SiHa cells transfected with hsa_circ_0018289 siRNAs or control. **(B)** Relative migration value of HeLa and SiHa cells transfected with hsa_circ_0018289 siRNAs or control. **(C)** Representative invasive images of HeLa and SiHa cells. **(D)** Relative invasion value of HeLa and SiHa cells. Data was represented as mean ± SEM. **P<0.01 represents statistical differences calculated.

It had been verified that hsa_circ_0018289 knockdown could inhibit cervical cancer cells proliferation in HeLa and SiHa cells *in vitro*. To further investigate the role of hsa_circ_0018289 on tumor physiological characteristics, transwell assay was performed in HeLa and SiHa cells *in vitro*. Results showed that hsa_circ_0018289 knockdown could suppress the migration (Figure 4A-4B) and invasion (Figure 4C-4D) vitality compared to control group, suggesting the suppressive role of hsa_circ_0018289 knockdown on cervical cancer cells aggression.

### Hsa_circ_0018289 knockdown inhibited tumor growth *in vivo*

**Figure 5:**
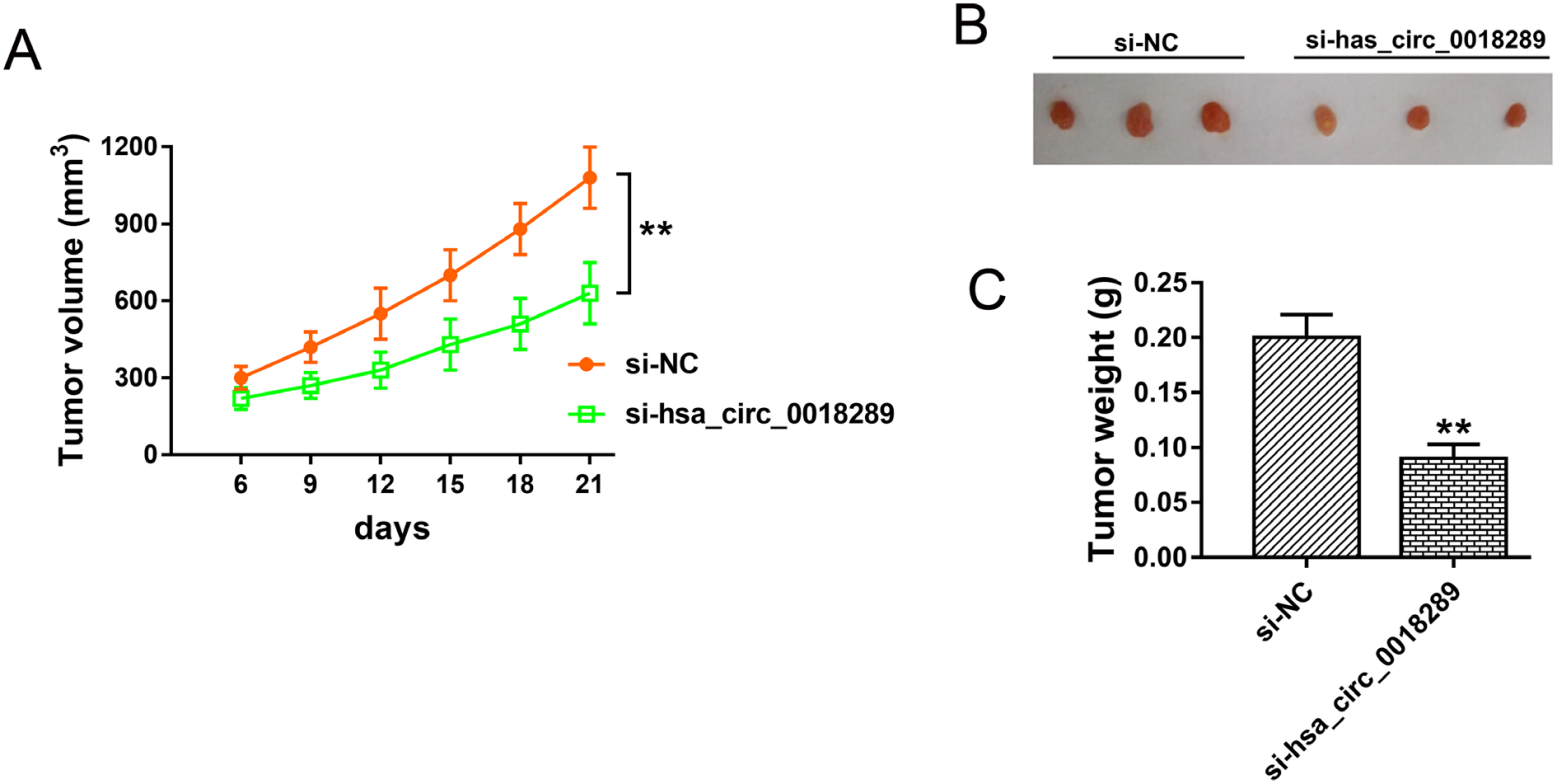
Hsa_circ_0018289 knockdown inhibited tumor growth *in vivo* **(A)** Tumor volumes and size of subcutaneous implantation mice models of HeLa cells stably transfected with si-hsa_circ_0018289 or controls. **(B)** The resected neoplasm from mice models, including si-NC (left) and hsa_circ_0018289 knockdown (right). **(C)** Tumor weight of control group and hsa_circ_0018289 knockdown group. Data was represented as mean ± SEM. **P<0.01 represents statistical differences calculated.

To investigate the role of hsa_circ_0018289 on tumor growth, xenograft model experiments were performed *in vivo*. HeLa cells stably transfected with si-hsa_circ_0018289 or empty were subcutaneously injected into flank of nude mice. The neoplastic volume was measured every 3 days after injection, and the weight was measured after three weeks. Results showed that hsa_circ_0018289 knockdown markedly decreased the tumor volumes and weights compared to the control group (Figure 5A-5C), suggesting the suppression of hsa_circ_0018289 knockdown on tumor growth *in vivo*.

### Hsa_circ_0018289 acted as ‘sponge’ and interacted with miR-497

**Figure 6:**
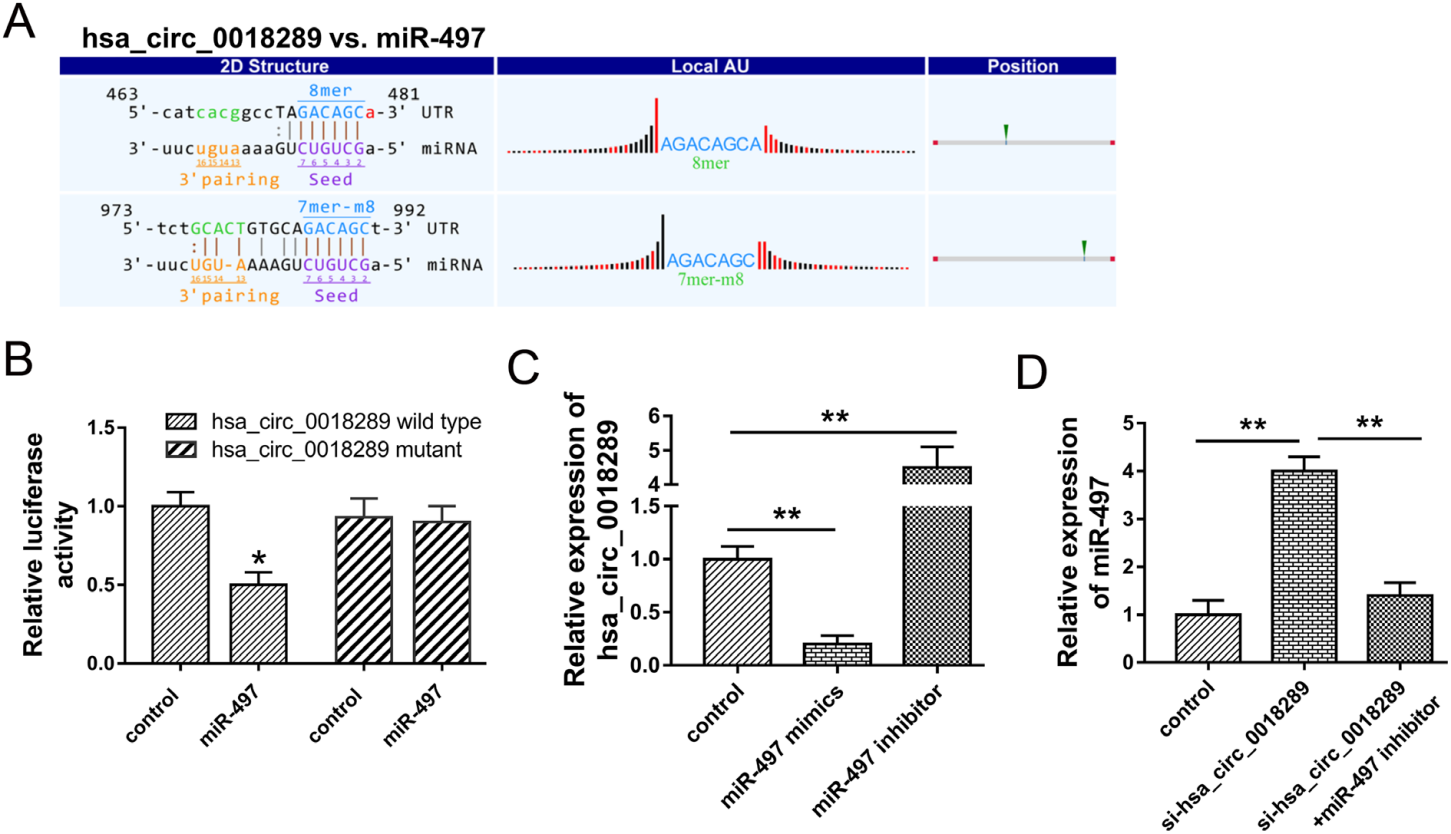
Hsa_circ_0018289 interacted with miR-497 **(A)** The putative target miR-497 for hsa_circ_0018289 predicted using Arraystar’s home-made software. **(B)** Luciferase reporter assay validated the interaction within hsa_circ_0018289 and miR-497. **(C)** Expression level of hsa_circ_0018289 in HeLa cells transfected with miR-497 mimics or inhibitor. **(D)** Expression level of miR-497 in HeLa cells transfected with si-hsa_circ_0018289 and/or miR-497 inhibitor. Data was represented as mean ± SEM. **P<0.01, *P<0.05 represents statistical differences calculated.

Presently, the role of circRNAs in tumor progression was still non-completely identified. The major admissive function of circRNAs was miRNAs ‘sponge’. The possible target miRNAs for hsa_circ_0018289 was predicted using Arraystar’s home-made software (Figure 6A). Luciferase reporter assay validated the combination within hsa_circ_0018289 and miR-497, showing the decreasing of luciferase intensity when hsa_circ_0018289 vector combined with miR-497 (Figure 6B). HeLa cells transfected with miR-497 mimics or inhibitor showed the decreased or increased expression levels of hsa_circ_0018289 (Figure 6C). Moreover, the co-transfection of si-hsa_circ_0018289 and miR-497 inhibitor could decrease the miR-497 expression compared to hsa_circ_0018289 knockdown group (Figure 6D). Results indicated that hsa_circ_0018289 interacted with miR-497, suggesting the miRNA ‘sponge’ role and the potential downstream targets of hsa_circ_0018289 in cervical cancer tumorigenesis.

## DISCUSSION

Presently, the role and mechanism of circRNAs in tumor progression are still not well identified [15]. Although circRNAs have been discovered for decades, the significance of circRNAs is recognized with the developing of high-throughput RNA-sequencing (RNA-Seq) and bioinformatics analysis [16, 17]. Emerging evidences have illustrated that circRNAs play important role in multiple physiological and pathological functions, involving cardiovascular system disease, nervous system disease and series of tumors [18–20]. Up to now, this is the first time to screen the circRNAs expression profiles in cervical cancer tissue, providing a vital insight for the regulation of circRNAs on cervical cancer genesis.

In present study, our team revealed the circRNAs expression profiles using human circRNAs microarray analysis in 4 pairs of cervical cancer tissue and adjacent noncancerous tissue. Total of 393 dysregulated circRNAs with 2 fold change were screened, and we further identified 45 significantly expressed circRNAs with 4 fold change, including 19 downregulated and 26 up-regulated. Among these up-regulated circRNAs, hsa_circ_0018289 was validated to be significantly up-regulated in 35 pairs of cervical cancer tissue and adjacent normal tissue. These aberrantly expressed circRNAs might participate in cervical cancer tumorigenesis and play important role for tumor occurrence and development.

Acting as a crucial member of ncRNAs family, circRNAs have been testified to exert more and more important role in multiple tumorigenesis [21]. For instance, circRNAs ciRS-7 (Cdr1as) expression level is much more upregulated in HCC than that in matched non-tumor tissues, and significantly correlated with hepatic microvascular invasion, acting as a promising biomarker and a novel therapy target for HCC [11]. In our study, loss-of-function experiments were performed to investigate the biological functions of hsa_circ_0018289 in cervical cancer tumor characteristics. Results revealed that hsa_circ_0018289 knockdown could suppress cervical cancer cells proliferation, migration and invasion *in vitro*. Furthermore, xenograft model experiments *in vivo* indicated that the tumor growth was inhibited in the hsa_circ_0018289 knockdown group, injected with stably transfected si-hsa_circ_0018289 HeLa cells, comparing to that in control group. In summary, loss-of-function experiments *in vitro* and *in vivo* collectively indicated the inhibition of hsa_circ_0018289 knockdown on cervical cancer proliferation and metastasis, suggesting the oncogenic role of hsa_circ_0018289 on cervical cancer growth.

In the research of circRNAs, high-throughput RNA sequencing and microarray analysis exert more and more important roles on the expression profilesscreening, and thousands of aberrantly expressed circRNAs have been reported [22]. In the initial stage, the focus of circRNAs research mainly concentrates on the expression profiles detection, for example, circRNA microarray discovers and identifies 23 upregulated and 48 downregulated circRNAs in basal cell carcinoma, describing a variety of potentially circRNAs involved in the molecular pathogenesis of BCC [23]. In radioresistant esophageal cancer cells, high-throughput sequencing detected 3752 candidate circRNA genes, containing 57significant upregulated circRNAs and 17 downregulated circRNAs, revealing a comprehensive expression and functional profile of differentially expressed circRNAs [24]. For cervical cancer tissue, our study firstly revealed the differently expressed circRNAs profiles, and showed the potential functional circRNAs in the cervical cancer carcinogenesis.

CircRNAs are a type of non-protein coding RNAs consisting of a circular loop with multiple miRNA binding sites, being called as miRNA response elements (MREs) and functioning as miRNA sponges. Until now, the major canonical function of circRNAs is miRNAs ‘sponge’, as well as lncRNAs. Because circRNAs have a specific covalently closed circular construction, it might harbor numerous miRNAs binding sites, acting as a huge ‘sponge’ to consume target miRNAs. For example, CDR1as (ciRS-7) comes from “circular RNA sponge for miR-7” with near 70 miR-7 binding site in the loop [25]. Moreover, in oral squamous cell carcinomas, circRNA_100290 regulate CDK6 expression through sponging miR-29b family members [26]. In present study, hsa_circ_0018289 was observed to directly bind to miR-497, which was validated by luciferase reporter assays. Although the further functional experiments are absent, the interaction within hsa_circ_0018289 and miR-497 could indicate the sponge role of hsa_circ_0018289 to miR-497.

Another important role of circRNAs is to act as biomarker for early detection in series of tumors. Due to the conservative covalently closed circular structure, circRNAs could resist the digestion of RNA enzyme, making its enrichment in peripheral blood or body fluid. For instance, the aberrant expression of hsa_circRNA_103636 in peripheral blood mononuclear cells is tested to be a potential novel biomarker for the diagnosis and treatment of major depressive disorder [27]. For cervical cancer, hsa_circ_0018289 is significantly up-regulated in tumor tissue and cells, providing a potential biomarker for cervical cancer patients’ early detection.

In summary, our study reveals the circRNAs expression profiles in cervical cancer tissue and identifies the functional candidate hsa_circhsa_circ_0018289 for cervical cancer tumorigenesis, suggesting the important suppressive role of hsa_circhsa_circ_0018289 knockdown on proliferation. These results provide a novel insight of circRNAs for cervical cancer carcinogenesis.

## MATERIALS AND METHODS

### Clinical specimens

A total of 35 pairs of cervical cancer tissue and matched non-tumor tissue were collected for the study in the Cangzhou Central Hospital and Zibo Central Hospital between Dec 2015 and Aug 2016. None of the cervical cancer patients received chemotherapy or radiotherapy before surgery or biopsy. All tissue samples were rapidly stored at −80°C after resection. This study was approved by the Ethics Committee of Cangzhou Central Hospital and Zibo Central Hospital. All the enrolled patients have signed the informed consent.

### CircRNA microarray analysis

Four pairs of cervical cancer tissue and matched non-tumor tissue samples were selected for microarray studies. RNA extraction and microarray hybridization were performed based on the Arraystar’s standard protocols. In briefly, total RNA was digested with Rnase R (Epicentre, Madison, USA) to remove linear RNA and enrich circular RNA. Then, RNAs were amplified for cRNA and labeled with an Arraystar Super RNA Labeling Kit (Arraystar, Rockville, the USA). Finally, these labeled RNAs were hybridized using Arraystar mouse circRNA Array (V1.0, Arraystar), and scanned by the Agilent Scanner G2505C.

### Cervical cancer cells and culture

Cervical cancer cells (HeLa, CaSki, SiHa, HT-3 and C33A), human epidermal cell (HaCaT) were purchased from the American Type Culture Collection (ATCC, Rockville, MD, USA). Cervical cancer cells were cultured in Dulbecco's modified Eagle medium (DMEM, Invitrogen, Carlsbad, CA, USA) supplemented with 10% FBS, L-glutamine (2 mM), 100 mg/ml penicillin and 100 mg/ml streptomycin (Invitrogen, Carlsbad, CA, USA). All cells were grown at 37°C in a cell incubator with a humidified atmosphere containing 5% CO_2_. Cells were transfected with indicated nucleotides or plasmid using Lipofectamine 2000 (Invitrogen, CA, USA) according to manufacturer’s instructions.

### Quantitative real-time PCR

Total RNA were isolated from cervical cancer tissues and cells using Trizol reagent (Invitrogen, Carlsbad, Calif, USA). Then, cDNA were synthesized using RevertAid First Strand cDNA Synthesis kit (Thermo Fisher Scientific, USA). Quantitative RT-PCR was performed using the SYBR-Green PCR Master Mix kit (Takara, Dalian, China). GAPDH acted as the endogenous control. All specific primers for circRNAs and miRNA were purchased from Sangon Biotech (Shanghai, China). The primer sequences were shown as follows: hsa_circ_0018289 (outward facing primers): 5’-TCACCAACCTTTGCCCTTCACACCT-3’, and 5’-AAGACTTACGTCTGTGTGCGTTGT-3’; miR-497, forward, 5’-CTCTTGAACTGCAGACTCA-3, reverse, 5’-TATGACATTTCAAGAATT-3’; GAPDH, forward, 5’-TCGACAGTCAGCCGCATCTTCTTT-3’, reverse, 5’-ACCAAATCCGTTGACTCCGACCTT-3’. Relative levels of gene expression were normalized to GAPDH housekeeping genes and calculated using the 2^-ΔΔCt^ method.

### Cell proliferation assay

Cell count kit-8 (CCK-8, Dojindo, Japan) was used to detect cell proliferation. Briefly, 3×10^4^ cervical cancer cells (HeLa and SiHa) were seeded into 96-well plates and 10 μl CCK-8 solution was added to each well. Then, the cells were incubated at 37°C for 90 minutes. At the indicated time points, the absorbance at 450 nm was measured using a spectrophotometer. The data are representative of three individual experiments carried out in triplicate.

### Migration and invasion assay

Transwell assay was performed for cervical cells (HeLa and SiHa) migration and invasion. In briefly, the inserts were coated with 50 μL Matrigel (BD Biosciences, Franklin Lakes, NJ, USA). Cells (5×10^4^) were suspended in 100 μl serum-free medium and then seeded on the upper floor of Transwell chambers (BD Biosciences, Franklin Lakes, NJ, USA). The lower chamber was added 500 μl serum with 20% FBS. After 48 h of incubating at 37°C with 5% CO_2_, the un-invaded cells were wiped with a cotton swab, and invaded cells were fixed in methanol and stained with 0.1% crystal violet. The number was counted under microscope. Each experiment was performed in triplicate.

### Bioinformatics analysis and luciferase reporter assay

The circRNA-miRNA interaction was predicted using Arraystar’s home-made miRNA target prediction software based on TargetScan and miRanda. For luciferase reporter assay, hsa_circ_0018289 cDNA was amplified and cloned into the downstream of the firefly luciferase gene pGL3 (Invitrogen, Carlsbad, Calif, USA). Then, HEK293T cells were co-transfected with wild type vector (150 ng) or mutant vector (150 ng). Besides, miR-497 mimics or miR-NC (2 ng) were also transfected into HEK293T cells using Lipofectamie 2000 (Invitrogen). After 48 h of transfection, the luciferase activities were detected using dual-luciferase reporter assay kit (Promega) normalized to Renilla luciferase activity. All the experiments were performed in triplicate.

### Xenograft mouse model

The xenograft mouse models were performed in nude mouse to determine the tumorigenicity. The animal assay was approved by the Institutional Committee of Cangzhou Central Hospital and Zibo Central Hospital and carried out based on the Institutional Animal Care and Use Committee’s guidelines. Male BALB/c nude mice (6 weeks) were purchased from Slac Laboratory Animal Center (Shanghai, China) and maintained under specific pathogen-free conditions. HeLa cells (5×10^6^ cells in 100 μl) transfected with si-hsa_circhsa_circ_0018289 were subcutaneously injected into the back of nude mice. The tumour size was measured every 3 days. At indicated times, the mice were sacrificed and tumor weight were measured.

### Statistical analysis

All statistical analysis in this study were performed by the Statistical Product and Service Solutions (SPSS) 16.0 software package (IBM, Chicago, IL, USA) and GraphPad Prism 6.0 (GraphPad Software, La Jolla, CA, USA). Paired t test, independent t test and one way analysis of variance (ANOVA) were used in this study correctly. P value of 0.05 or less was considered statistically significant.
